# Genome-wide characterization and expression divergence of voltage-dependent anion channel (VDAC) in upland cotton (*Gossypium hirsutum* L.) in response to abiotic stresses

**DOI:** 10.3389/fpls.2025.1682305

**Published:** 2025-09-30

**Authors:** Umar Akram, Ali Ahmad, Muhammad Nadeem Shah, Furqan Ahmad, Muhammad Baber, Muhammad Tanveer Altaf, Muhammad Aneeq Ur Rahman, Önder Albayrak

**Affiliations:** ^1^ Institute of Plant Breeding and Biotechnology, Muhammad Nawaz Shareef University of Agriculture Multan, Multan, Pakistan; ^2^ Institute of Molecular Biology and Biotechnology, Bahauddin Zakariya University, Multan, Pakistan; ^3^ Indiana Wesleyan University, Merrillville, IN, United States; ^4^ Department of Agriculture, Government College University Lahore, Lahore, Pakistan; ^5^ Department of Field Crops, Faculty of Agriculture, Recep Tayyip Erdoğan University, Rize/Pazar, Türkiye; ^6^ Biotechnology Research Institute, Graduate School of Chinese Academy of Agricultural Sciences, Beijing, China

**Keywords:** voltage-dependent anion channels (VDACs), *G. hirsutum*, phylogeny, stress response, motif

## Abstract

Voltage-dependent anion channels *(VDACs)* are proteins present at the outer membrane of mitochondria that are involved in stress response, such as drought and salt, signal transduction, and cellular metabolism. The role of the *VDAC* gene family in other crops has been identified, but its role in cotton has been uncharacterized. In this study, a genome-wide study was conducted in upland cotton (*G. hirsutum* L.) under drought and salt stresses. We identified 18 *GhVDAC* genes and mapped them using the MG2C online tool. The random distribution of *GhVDAC* genes was determined using the GSDS server for their intron-exon structure. Phylogenetic analysis using MEGA. 11 software constructed the phylogenetic tree by the maximum likelihood method of 14 different species with 66 *VDAC* genes, which were distributed across four clades. All *p. patens VDAC* genes were present in a single clade, and 10 *GhVDAC* genes were found in clade IV. Ten motifs were identified, and motif 9 was absent in *GhVDAC3, GhVDAC9, GhVDAC10, GhVDAC11*, and *GhVDAC18.* Motif 10 was present in only two genes, *GhVDAC5* and *GhVDAC14.* Promoter analysis revealed cis-elements involved in hormone and stress response. Synteny and circos analysis revealed orthologous gene duplication events and evolutionary relationships. In-silico expression mapping was used to identify candidate genes, and then qRT-PCR was done for validation of *GhVDAC6, GhVDAC11, GhVDAC13*, and *GhVDAC15* genes. In conclusion, this is a genome-wide study on the *VDAC* gene family in cotton under drought and salt stress that provides insights into their structure, evolutionary relationship, and molecular mechanism, and this study lays the theoretical foundation for future breeding programs to develop resistant crops for drought and salt stress.

## 1 Introduction

*Gossypium hirsutum* L. is a widely cultivated cotton crop variety that produces 90% of the fiber among all cotton species (Li et al., 2022). Due to harsh environmental conditions, the yield and quality of cotton crop need to be improved (Liu et al., 2021). Almost 60 different countries produce cotton around the globe, and it is the third-largest crop after canola and soybeans. After China, India, the USA, and Brazil, Pakistan is the 5^th^ largest producer of cotton in the world that accounting for about 40% of the industrial sector accounts cotton (Raza et al., 2023; Khan et al., 2024). Cotton production has always been hindered due to several environmental stresses, such as salt, drought, inadequate water resources, and high temperatures, but salinity and drought stresses are the major stresses that influence the yield (Naresh et al., 2024).

As the population is increasing, there is a need to produce crops globally, as the crop production demand has increased at least by 40% in arid and semi-arid areas (Pennisi, 2008). But climate change is the biggest challenge for crop production, which can lead to severe abiotic and biotic stresses (Nakashima et al., 2014; Shaar-Moshe et al., 2017). Salt stress affects almost one billion hectares of semi-arid and dry regions around the world, and it is responsible for low cotton yield, which decreases production by about 50-60%. Under salt stress, the Na^+^/Cl level increases and disrupts the natural defense system of plants (Munns and Tester, 2008; Jain et al., 2024). On the other hand, water is essential for every crop at every stage, from seed germination to maturity. The terminology “drought” refers to the reduced groundwater and rainfall availability with the rise in temperature. When crops are exposed to drought stress, several parameters are disrupted, such as reduced photosynthesis, cell shrinkage, and leaf rolling, which ultimately lead to wilting, thus leading to plant death (Joshi et al., 2016). Drought can cause anatomical, physiological, and morphological disturbance among all crops especially in cotton, which results in lower yield (Farooq et al., 2008; Saleem et al., 2016; Haj Sghaier et al., 2022). In response to drought stress, plants regulate gene expression via complex transcriptional networks (Singh and Laxmi, 2015). Thus, it is important to develop transgenic stress-resilient crops to sustain crop production globally.

Voltage-dependent anion-selective channel *(VDAC)* genes are mitochondrial proteins that comprise almost 30% of the mitochondrial area in eukaryotic organisms. The *VDAC* genes are involved in environmental and developmental stresses such as drought and salt (Takahashi and Tateda, 2013; Ashraf et al., 2021). To maintain homeostasis during drought and salt stress, these genes regulate the transport of metabolites. The *VDAC* proteins interact with several other proteins for gene regulation in mitochondria and the cytoplasm (Gonçalves et al., 2007). Reactive oxygen species (ROS) can be induced via high accumulation of ROS pathways under abiotic stresses, which leads to programmed cell death (PCD) due to the high accumulation of ROS (Ying et al., 2008; Suzuki et al., 2011; Dietz et al., 2016; Nadarajah, 2020; Sohaib et al., 2023). An enzyme, nucleoredoxin 1, is a member of the thioredoxin family, is responsible for the to protect the detoxifying enzymes from oxidative stress (Mittler, 2002). The recent *VDAC* gene study on Arabidopsis showed that *AtVDAC3* and thioredoxin are responsible for the regulation of ROS in plants (Zhang et al., 2015). Hence, this PCD, due to mitochondrial-mediated apoptosis, is also due to the *VDAC* genes (Yu et al., 2022).

Several genes, such as *CDPK* (Shi and Zhu, 2022), *NHX* (Akram et al., 2020), *Box* (Sun et al., 2023), SAMS (Kilwake et al., 2023), *BASS* (Myo et al., 2021), *GDSL* (Liu et al., 2023), and many more, are involved in different abiotic stresses in the cotton plant. Moreover, there are few studies on the *VDAC* gene family, like in tobacco (Yang et al., 2021), china rose (Ashrafei Kolteh et al., 2022), wheat (Yu et al., 2022), rice (Xu et al., 2015), beans (Saidani et al., 2016), pearl millet (Desai et al., 2006), and Arabidopsis (Yu et al., 2022), but still, the *VDAC* gene family in *G. hirsutum* L. remains unexplored.

In the current study, a genome-wide characterization study of the *VDAC* gene family in cotton under salt and drought stresses was performed. The PF01459 Pfam domain was used to identify candidate *GhVDAC* sequences to performvarious bioinformatic analyses, including subcellular localization, phylogenetic analysis, Protein-Protein Interaction (PPI), motif analysis, promoter analysis, synteny and circos analysis, in-silico expression analysis, and qRT-PCR. These analyses provide a better molecular function of the *VDAC* gene.

## 2 Materials and methods

### 2.1 Identification and sub-cellular localization of *GhVDAC* genes

The genomic, conserved domain sequences (CDS), and amino acid sequences were retrieved from the CottonMD database (https://yanglab.hzau.edu.cn/CottonMD.1) (Yang et al., 2023) after performing protein BLAST (BLASTP), and protein arabidopsis sequences were used as a query. *GhVDAC* genes with a score of >100 and an e-value of <10*^−^* ^5^ were selected as candidate cotton genes for further analyses. HMMER 3.0 (https://www.ebi.ac.uk/Tools/hmmer/) (Finn et al., 2011) was used for the identification of *VDAC* genes in cotton by using PF01459 as a Pfam domain after confirming it through the SMART website (http://smart.embl-heidelberg.de) database (Letunic et al., 2021). The redundant sequences were removed from the files, and the obtained sequences were converted into FASTA format. The physiochemical properties of *GhVDAC* genes were extracted from the Expasy database (https://web.expasy.org/protparam/) (Gasteiger et al., 2003). Sub-cellular localization of *GhVDAC* genes was predicted through the WoLFPSORT database (https://wolfpsort.hgc.jp/) (Horton et al., 2007). All the parameters were set to default.

### 2.2 Chromosomal mapping, chromosome linkage, and gene structure analysis of *GhVDAC* genes

The exon-intron boundaries of *GhVDAC* genes were visualized using the Gene Structure Display Server (GSDS) (http://gsds.cbi.pku.edu.cn) (Hu et al., 2015) and MG2C (http://mg2c.iask.in/mg2c_v2.1/) databases (Chao et al., 2021), respectively.

### 2.3 Phylogenetic tree construction and visualization analysis

For constructing a phylogenetic tree of *VDAC* genes across different plant species, the maximum likelihood method (ML) with 1,000 bootstrap method was employed in MEGA.11 software (Felsenstein, 1981; Kumar et al., 2008). The multiple sequence alignment via MUSCLE (Edgar, 2004) was done for several plant species such as *A. thaliana* (At) from TAIR database (https://www.arabidopsis.org/) (Lamesch et al., 2012)*, G. hirsutum* (Gh) and *G. barbadense* (Gb) from cottonmd database*, G. arboreum* (Ga) and *G. raimondii* from cottongen database (https://www.cottongen.org/) (Yu et al., 2014), *P. patens* (Pt)*, S. mollendorfii* (Sm)*, T. cocao* (Tc) and *T. aestivium* (Ta) Ensembl plant database (https://plants.ensembl.org/index.html) (Bolser et al., 2016)*, S. bicolor* (Sb)*, O. sativa* (Os) *A. comosus* (Ac)*, G. max* (Gm), and *Z. mays* (Zm) from phytozome database (https://phytozome-next.jgi.doe.gov/) (Goodstein et al., 2012). Furthermore, the iTOL online tool (https://itol.embl.de/) (Letunic and Bork, 2016) was used for circular visualization of the tree.

### 2.4 Motif, protein–protein interaction, and promoter analysis of *GhVDACs*

After setting the total number of motifs to 10, the motifs of *GhVDAC* genes were found using the MEME suite (https://meme-suite.org/meme/tools/meme) database (Bailey et al., 2009). To analyze the protein interaction of *GhVDAC* genes, the STRING database (https://string-db.org/) was used (Szklarczyk et al., 2021). The promoter analysis of *GhVDAC* genes was done by using the PlantCare database (https://bioinformatics.psb.ugent.be/webtools/plantcare/html/) (Lescot et al., 2002) to find out different stress-responsive genes.

### 2.5 Synteny and circos plot construction of *GhVDAC* genes

The TbTools software (Chen et al., 2020) was used to visualize *GhVDAC* genes on corresponding chromosomes. The synteny analysis was performed among *G. hirsutum, G. arboreum, G. raimondii*, and *A. thaliana* using TbTools software. The MCScanX with *≤* 1 *×* 10*^−^* ^5^ threshold e-value was used to calculate the duplication events (Wang et al., 2012).

### 2.6 Expression profiling and heatmap visualization

The in-silico expression data of drought and salt stress of *GhVDAC* genes were retrieved from the cottonmd database, and it was further confirmed by using the NCBI Geodataset (https://www.ncbi.nlm.nih.gov/bioproject/PRJNA490626 accessed on 1^st^ May, 2025). The expression heatmap using FPKM values was generated using TbTools.

### 2.7 Primer design, RNA, cDNA synthesis, and qRT-PCR analysis

The TRIzol method (Rio et al., 2010) was used for RNA extraction of the candidate gene after designing the primer, and then the Thermofisher cDNA kit was used for cDNA synthesis. The specificity was determined by using the melting curve, and a student’s t-test analysis was done with a P<0.05 significance.

### 2.8 Salt and drought stress treatments for *GhVDAC* genes

For drought and salt stress in *G. hirsutum*, the TM-1 (Texas marker) cultivar of cotton was grown in 12 hours of light and 12 hours of dark conditions under greenhouse conditions. For salt stress, the 400mM salt was applied to 2-week-old plant leaves. The drought stress was applied by withholding water till the relative water content (RWC) reached about 7% in pots. The controls for NaCl and drought treatment remained the same, and no stress was applied. The second and third true leaves were used for qRT-PCR analysis. The samples were kept at -80°C after being snap-frozen in liquid nitrogen. The results of both salt and drought stress were checked after 0 hours, 3 hours, 12 hours, and 24 hours.

### 2.9 qRT-PCR analysis of *GhVDAC* genes

For qRT-PCR, BioRAD qPCR analyzer was used, and the cycles that were used are 95 °C for pre-denaturation for 30 seconds, 95 °C for denaturation for 5 seconds, annealing/extension till fluorescent signal, for 60 °C for 1 minute. Three biological replicates were analyzed, and relative expression of *GhVDAC* genes was calculated by the 2^−△△Ct^ method (Livak and Schmittgen, 2001).

## 3 Results

### 3.1 Gene structure, subcellular localization, and chromosome mapping

**Figure 1:**
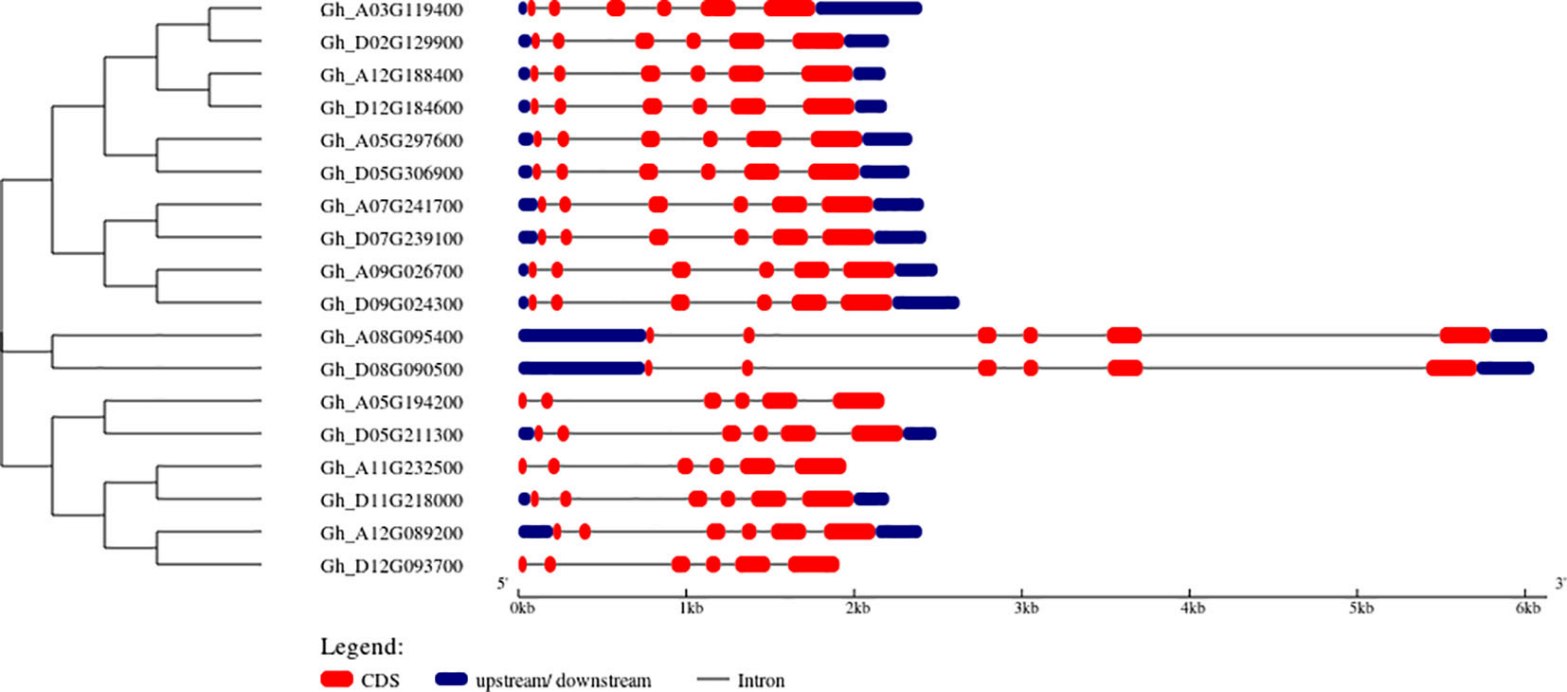
Structural representation of *GhVDAC* genes by using the GSDS server tool. The red color indicates the CDS region, and the black line indicates the intronic region. The Y-axis shows gene IDs in phylogenetic Tree order While the X-axis shows the length of genes in kilo base pairs (Kb).

The gene structure analysis through the GSDS server is represented by blue color boxes for upstream and downstream regions, a black line for introns, and red color boxes for CDS regions. The results of 18 *GhVDAC* genes are shown in **Figure 1**.

**Figure 2:**
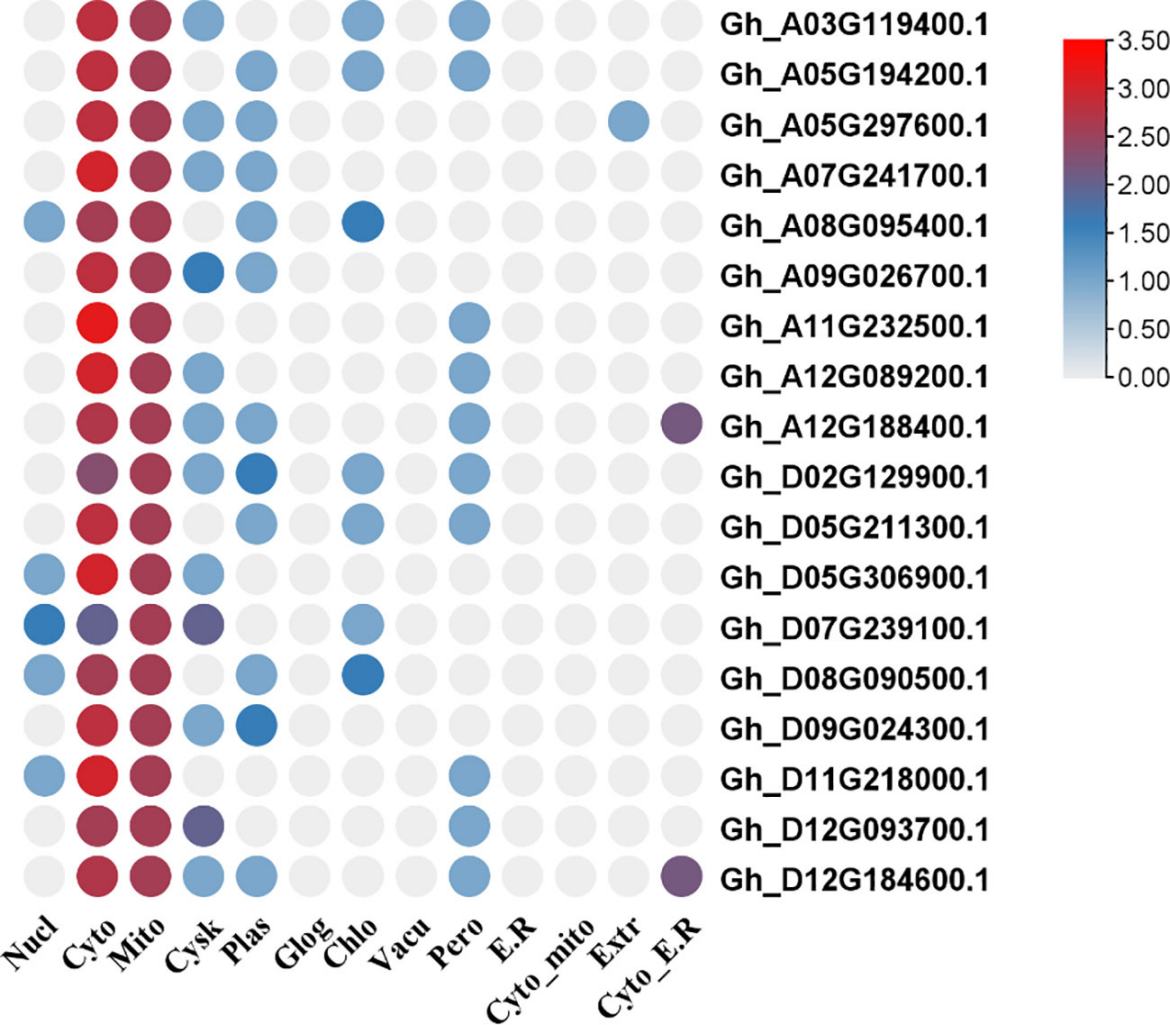
Sub-cellular localization of *GhVDAC* genes by using the WOLFPSORT database. The X-axis shows the cell organelle names while the Y-axis shows the gene IDs.

The transcript IDs, gene names, molecular weight (MW), amino acid (A.A.) numbers, theoretical isoelectric point (PI), grand average of hydropathy (GRAVY), and predicted subcellular locations are represented in **Table 1**. The diagrammatic representation of the predicted subcellular locations of *GhVDAC* genes is shown in **Figure 2**.

**Table 1:** Structural properties of *GhVDAC* genes.

Gene ID	Gene name	Molecular weight (M.w) (kDa)	Amino acid (A.A)	Theoretical PI (pH)	GRAVY	Sub-cellular localization
*Gh_A03G119400*	*GhVDAC1*	29656.34	276	7.06	-0.212	Cytoplasm
*Gh_A05G194200*	*GhVDAC2*	29043.98	273	9.15	-0.165	Cytoplasm
*Gh_A05G297600*	*GhVDAC3*	29637.48	276	8.56	-0.209	Cytoplasm
*Gh_A07G241700*	*GhVDAC4*	29381.16	276	8.79	-0.174	Cytoplasm
*Gh_A08G095400*	*GhVDAC5*	29502.71	273	8.79	-0.139	Cytoplasm + Mitochondria
*Gh_A09G026700*	*GhVDAC6*	29542.49	276	8.77	-0.178	Cytoplasm
*Gh_A11G232500*	*GhVDAC7*	28878.95	270	9.35	-0.154	Cytoplasm
*Gh_A12G089200*	*GhVDAC8*	29544.60	276	9.11	-0.155	Cytoplasm
*Gh_A12G188400*	*GhVDAC9*	29399.07	276	7.06	-0.191	Cytoplasm
*Gh_D02G129900*	*GhVDAC10*	29565.23	276	7.06	-0.192	Mitochondria
*Gh_D05G211300*	*GhVDAC11*	29332.16	276	8.90	-0.199	Cytoplasm
*Gh_D05G306900*	*GhVDAC12*	29603.50	276	7.10	-0.157	Cytoplasm
*Gh_D07G239100*	*GhVDAC13*	29397.16	276	8.79	-0.174	Mitochondria
*Gh_D08G090500*	*GhVDAC14*	29498.62	273	8.29	-0.148	Cytoplasm + Mitochondria
*Gh_D09G024300*	*GhVDAC15*	29370.33	276	8.88	-0.146	Cytoplasm
*Gh_D11G218000*	*GhVDAC16*	29563.66	276	9.28	-0.183	Cytoplasm
*Gh_D12G093700*	*GhVDAC17*	29565.23	276	7.06	-0.192	Cytoplasm + Mitochondria
*Gh_D12G184600*	*GhVDAC18*	29399.07	276	7.06	-0.191	Cytoplasm

**Figure 3:**
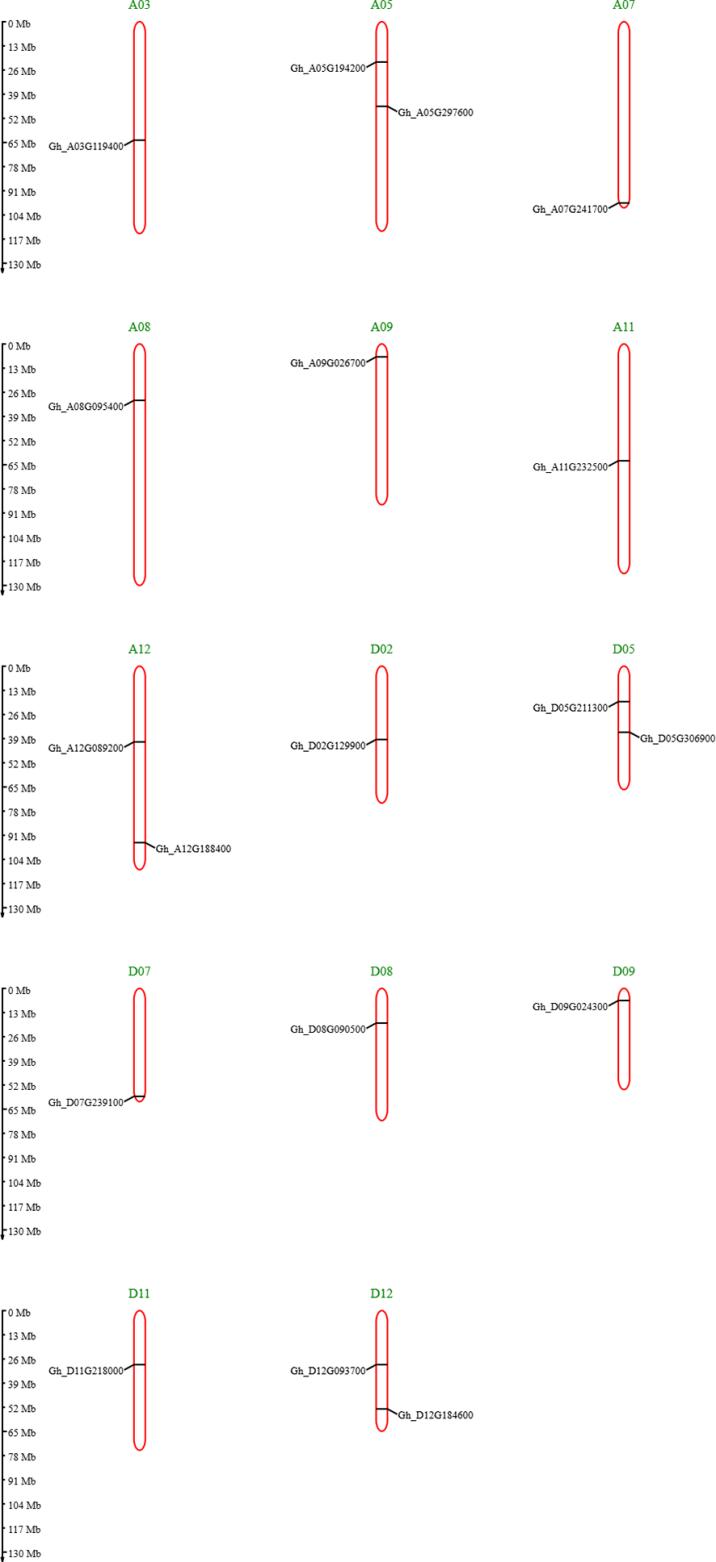
Chromosome mapping of *GhVDAC* genes by using the MG2C database.

The cytoplasm had most genes present, and some genes were present in both, cytoplasm and mitochondria. The chromosome mapping of genes shows that A05, A12, and D12 chromosomes had two genes present on them, whereas all remaining chromosomes had a single gene present (**Figure 3**). The detailed file of subcellular localization is in **Supplementary Table S1**.

### 3.2 Motif and sequence logos analysis

**Figure 4:**
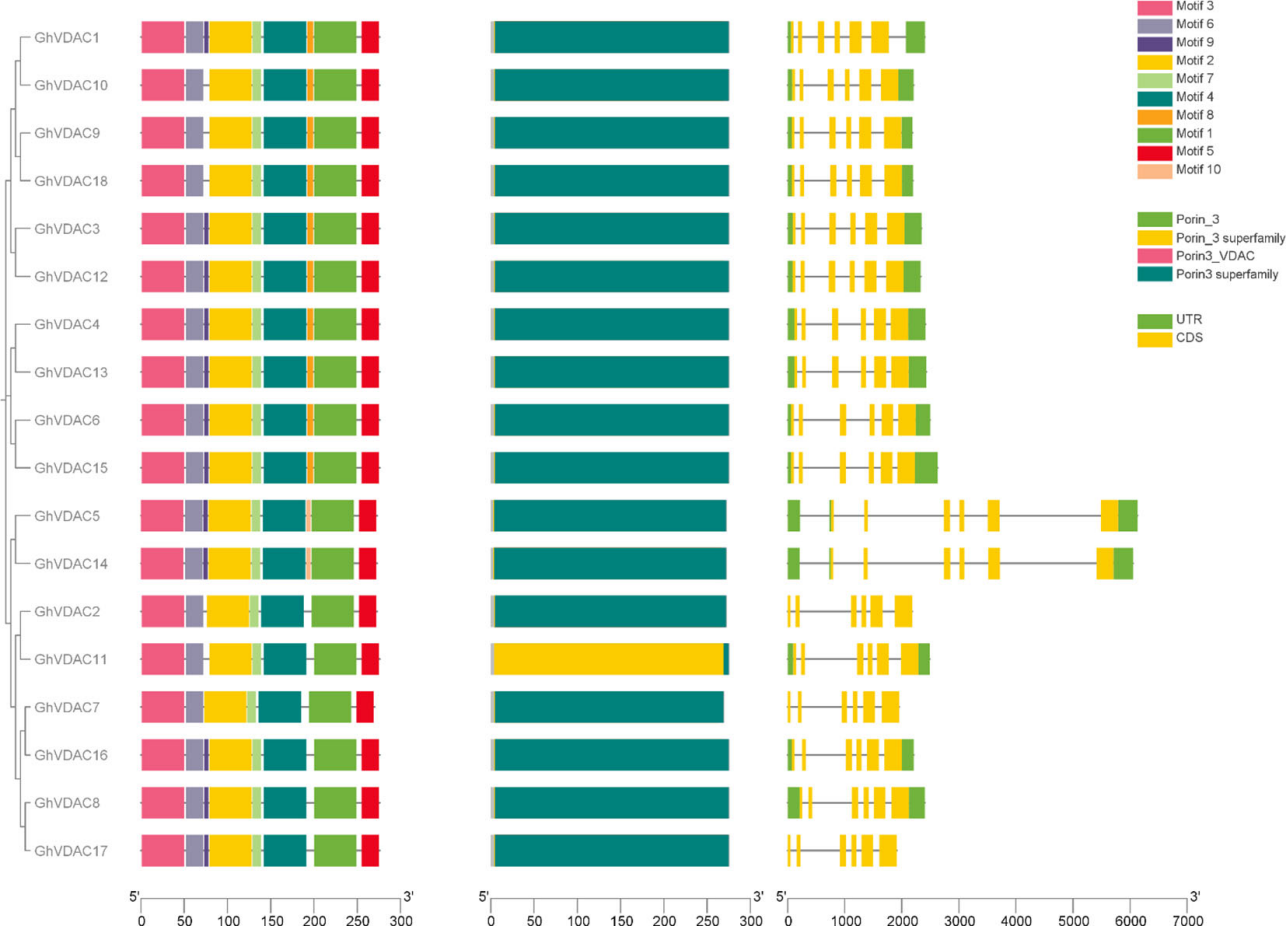
*GhVDAC* phylogenetic relationship, gene structure, and conserved motifs. The different motif boxes were present in different colors, numbered from 1 to 10. The exons are represented by Yellow boxes, and introns are represented by black lines. The X-axis represents the length and the Y-axis shows the Gene IDs in Phylogenetic tree order.

The *GhVDAC* gene motifs from the Meme Suite database were identified, and interestingly, among all domains, the porin3 superfamily was abundantly present in all motifs. Also, motif 9 was present in all genes except *GhVDAC2, GhVDAC7, GhVDAC9, GhVDAC10, GhVDAC11*, and *GhVDAC18*. Motif 10 was present in only two genes, *GhVDAC5* and *GhVDAC14*. Moreover, the UTRs were absent in *GhVDAC2, GhVDAC7*, and *GhVDAC17.* The results suggest that these genes may be involved in the stress regulatory mechanism (**Figure 4**). The file for motif analysis is present in **Supplementary Table S2**.

### 3.3 Phylogenetic tree construction of *VDAC* genes

**Figure 5:**
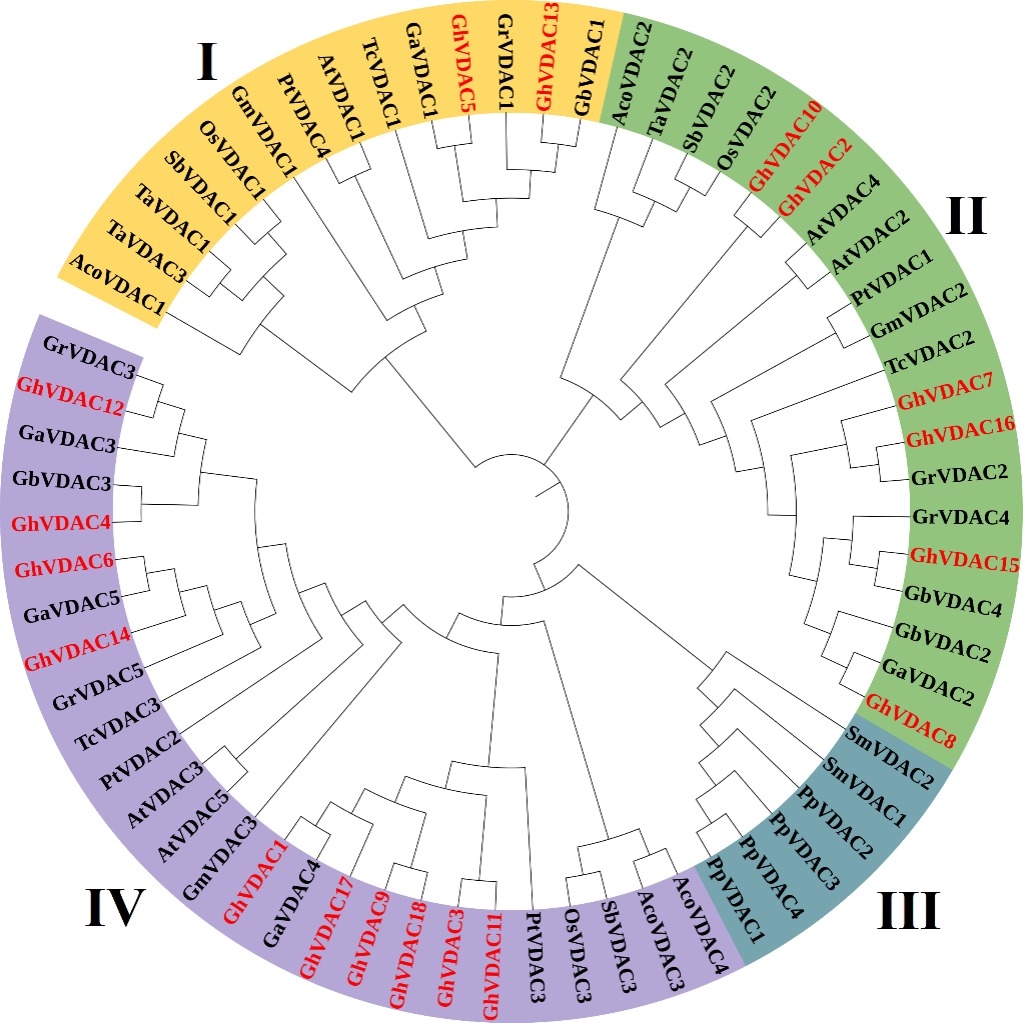
The phylogenetic tree of 14 different species was constructed using the maximum likelihood method (ML) with 1000 bootstrap replicates in MEGA 11. The phylogenetic tree was distributed into 4 clades, and *GhVDAC* was marked in red color.

The *VDAC* genes of several plant species were analyzed using MEGA software, and iTOL was used for circular visualization. The results indicated that a total of 66 genes were identified from 14 different plant species phylogenetic tree construction. Interestingly, all of the *G. arboreum* genes were present in clade I, and 10 genes of *G. hirsutum* were present in clade IV. Also, the *G. hirsutum* exhibited a close relation with *T. cacao* and other cotton species (**Figure 5**). The Sequences of all species are present in **Supplementary Table S3**.

### 3.4 Cis-regulatory element analysis of *GhVDAC*s

**Figure 6:**
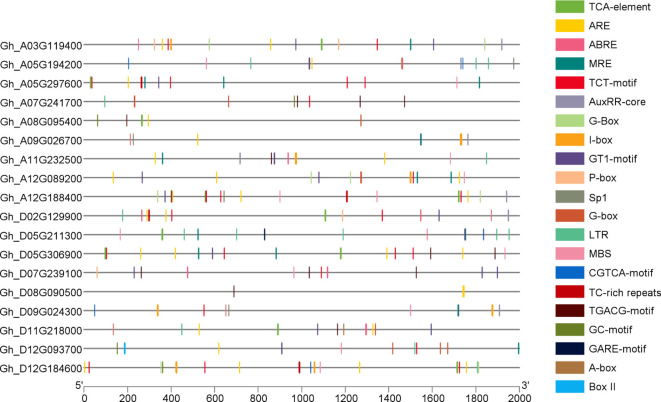
Distributions of different cis-acting elements in *GhVDAC* gene promoters. Different colors indicate the cis-elements. The -axis shows the length of the promoter region in base pairs, and Y-axis shows the GhVDAC gene IDs.

The promoter analysis of *GhVDAC* genes was done using the PlantCARE database with a 2 kb upstream region. The results indicated that several cis-elements were found in *GhVDAC* genes, such as MBS, GT-1 motif, G-box, ABRE, TCA-element, and LTR. Hormone-responsive cis-elements like TCA elements (salicylic acid), CGTCA motifs (jasmonic acid), exhibit distinct patterns, suggesting their involvement in hormonal signaling pathways; stress-related cis-elements, such as ABRE (abscisic acid response), MBS (MYB-binding site for drought), are distributed with stress-associated regions. The light-responsive element, like the G-box, played a role in photo-regulation (**Figure 6**). The raw cis-elements file of *GhVDAC* genes is provided in **Supplementary Table S4**.

### 3.5 Protein-protein interaction of *GhVDAC* genes

**Figure 7:**
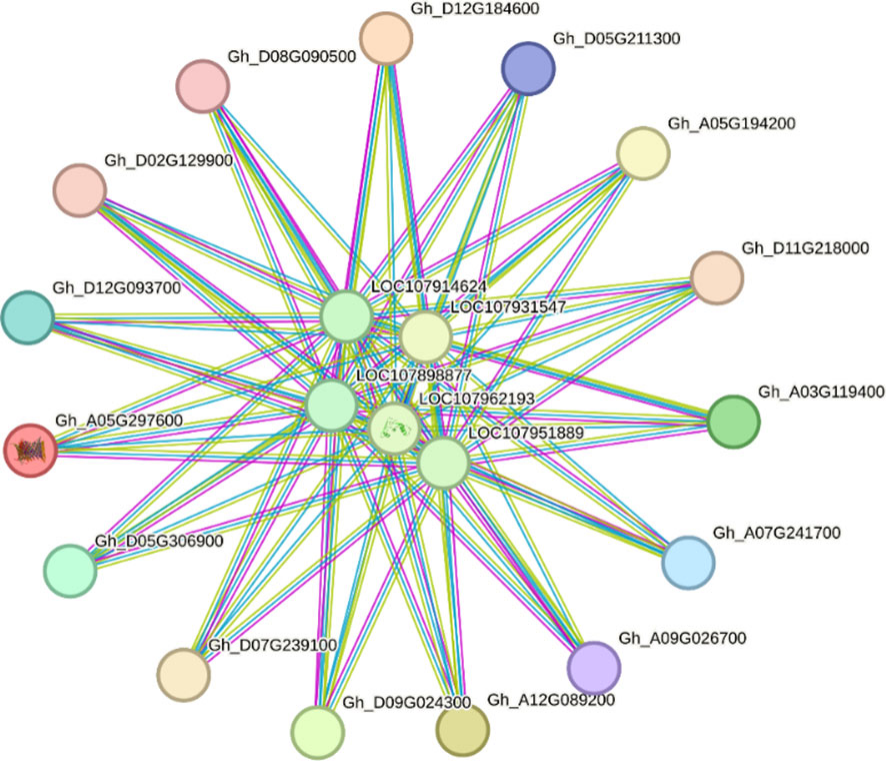
Protein-protein interaction (PPI) network of *GhVDAC* genes. Specific protein interaction of *VDAC* genes in *G. hirsutum* was determined by using the STRING database.

The string database was used for PPI of *GhVDAC* genes, and the analysis showed that the clustering coefficient was 0.597 with 20 nodes and 82 edges. The results indicated that, as a group, these proteins are partially biologically connected (**Figure 7**).

### 3.6 Ka/Ks ratio and collinearity analysis of *GhVDAC* genes

The selective pressure analysis was performed to understand the coding sequences for proteins. To check whether the selective pressure is acting upon them or not, the ratio between the non-substitution rate and protein-coding substitution rate was checked. The substitution mutations are not considered as natural selection because they do not disturb the protein function or structure. The non-substitution mutations, on the other hand, disrupt the protein functions. If Ka/Ks < 1, it is considered a negative selection; if Ka/Ks > 1, it is considered positive selection. Hence, our results indicated that all *GhVDAC* genes had Ka/Ks < 1, thus, indicating the negative selection in *GhVDAC* genes. The Ka/Ks ratio data of *GhVDAC* genes is provided in **Supplementary Table S5**.

**Figure 8:**
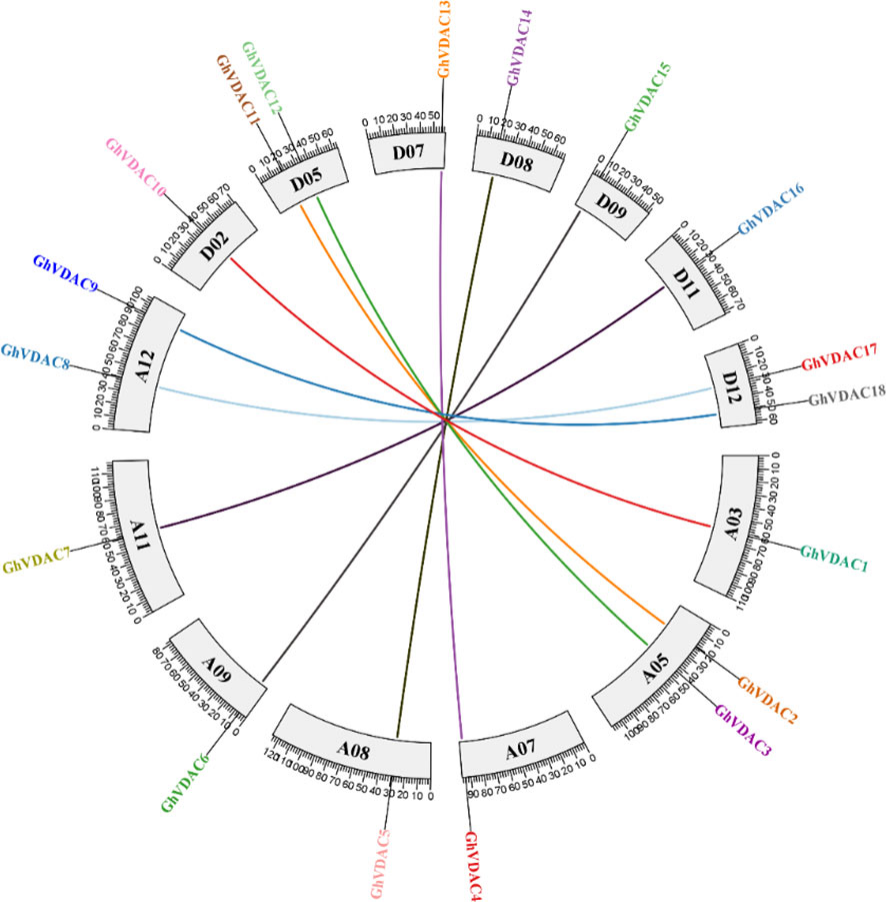
Collinearity analysis of all *GhVDAC* genes among all Gossypium chromosomes.

The segmental and tandem duplications are critical for gene family expansion, as segmental duplications are multiple chromosomal blocks present in a genome. On the other hand, tandem duplications are adjacent genes on the same chromosome. Our results suggested that both segmental and tandem duplications were found in collinearity analysis, as shown in **Figure 8**. The *GhVDAC* genes have Ka/Ks < 1, demonstrating that duplicated genes are purified proteins that are stable.

### 3.7 Synteny analysis of *GhVDAC* genes with other species

**Figure 9:**
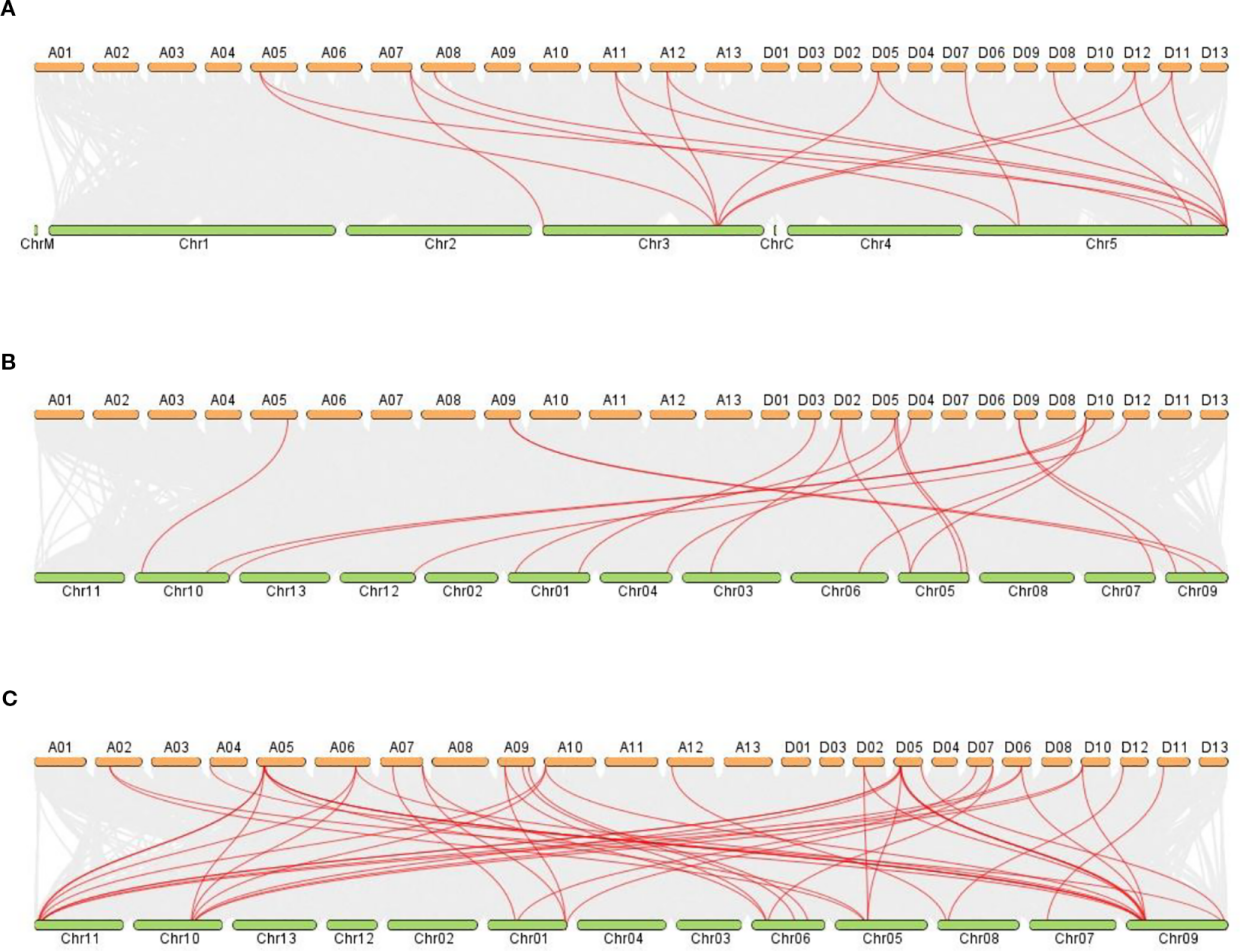
**(A–C)** The collinear relationship between *G*. *hirsutum* and other crops. **(A)** *G*. *hirsutum* vs *A*. *thaliana* **(B)** *G*. *hirsutum* vs *G*. *arboreum* C) *G*. *hirsutum* vs *G*. *raimondii*. Red lines indicate the collinear gene pairs of *G*. *hirsutum* and other crops, and grey lines represent the syntenic blocks. The X-axis shows the number of chromosomes present between two species.

The synteny analysis was performed between *G. hirsutum* and other plant species, like *G. raimodaii, G. barbadense, A thaliana*, and *G. arboreum* (**Figures 9A–C**). A total of 17 orthologous genes are dispersed in *G. hirsutum* vs *A. thaliana*, except A01-A10, A13, D01-D04, D06, D09, D10, D11, and D13 (**Figure 9A**). Also, on *G. hirsutum* and *G. arboreum*, 17 orthologous genes were identified on A05, A09, D02-D05, D09, D10, and D12 (**Figure 9B**). Interestingly, 41 orthologous genes were identified in *G. hirsutum* vs *G. raimondii*, except A01, A03, A08, A11, A13, D01, D03, D04, D08, and D13 (**Figure 9C**).

### 3.8 Heatmap analysis of *GhVDAC*s

**Figure 10:**
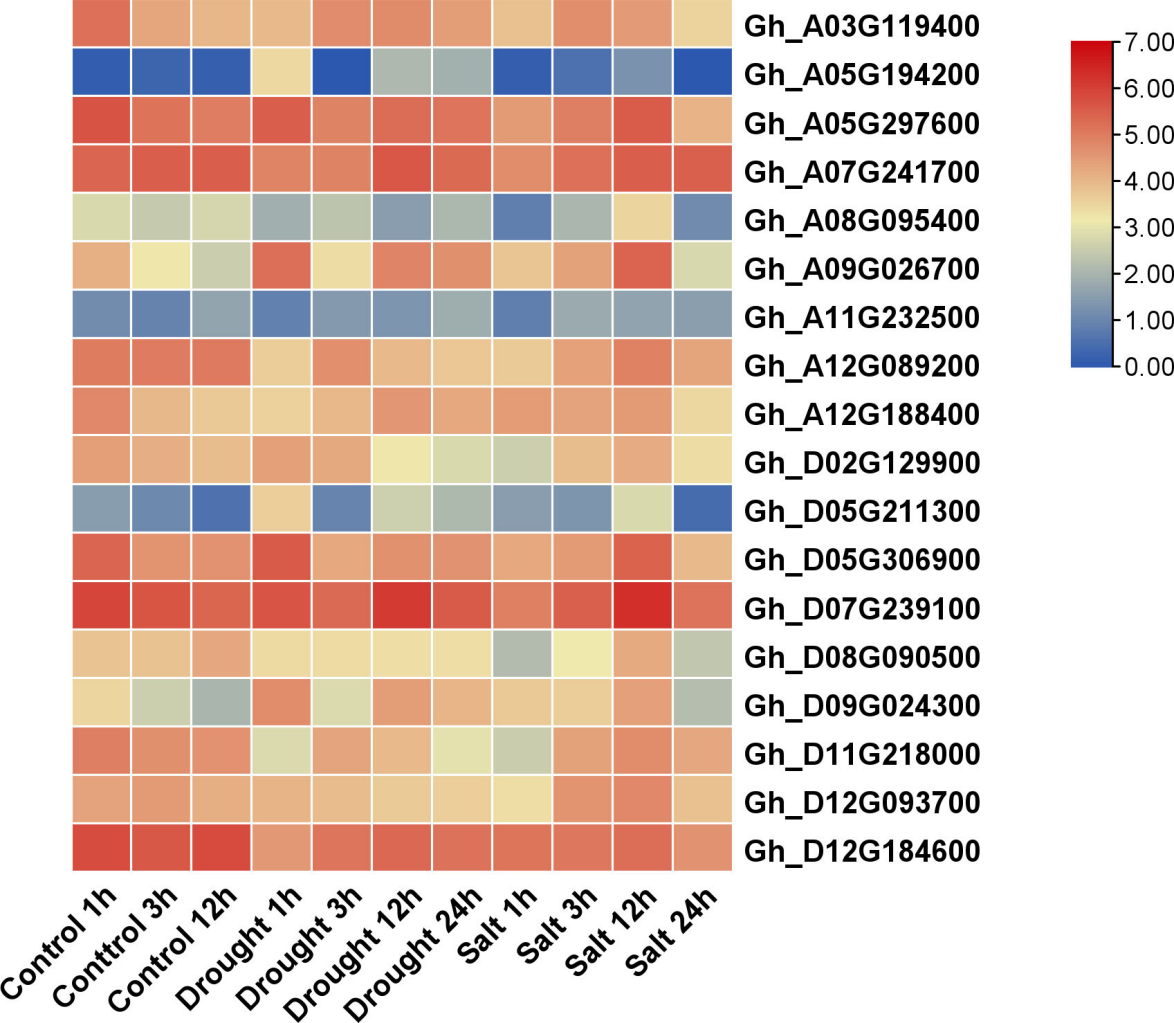
Expression profile of 18 *GhVDAC* genes under drought and salt stresses with logarithm with base 2 for standardization. Different colors represent the expression level of cotton leaves under stresses. The X-axis represents the different stress treatments with time intervals, and the Y-axis represents the Gene IDs.

The heatmap expression analysis of *GhVDAC* genes was performed using publicly available RNA-seq data. The control, drought, and salt stress data were obtained under 1 hour, 3 hours, and 12 hours for control, whereas 1 hour, 3 hours, 12 hours, and 24 hours for drought and salt stress. Out of the 18 *GhVDAC* genes, 4 candidate genes (*Gh_A03G06296, Gh_A09G01181, Gh_A07G13146*, and *Gh_A12G04801*) were identified on their in-silico expression pattern (**Figure 10**). These candidate genes were further submitted for qRT-PCR analysis.

### 3.9 qRT-PCR analysis of candidate *GhVDAC* genes

**Figure 11:**
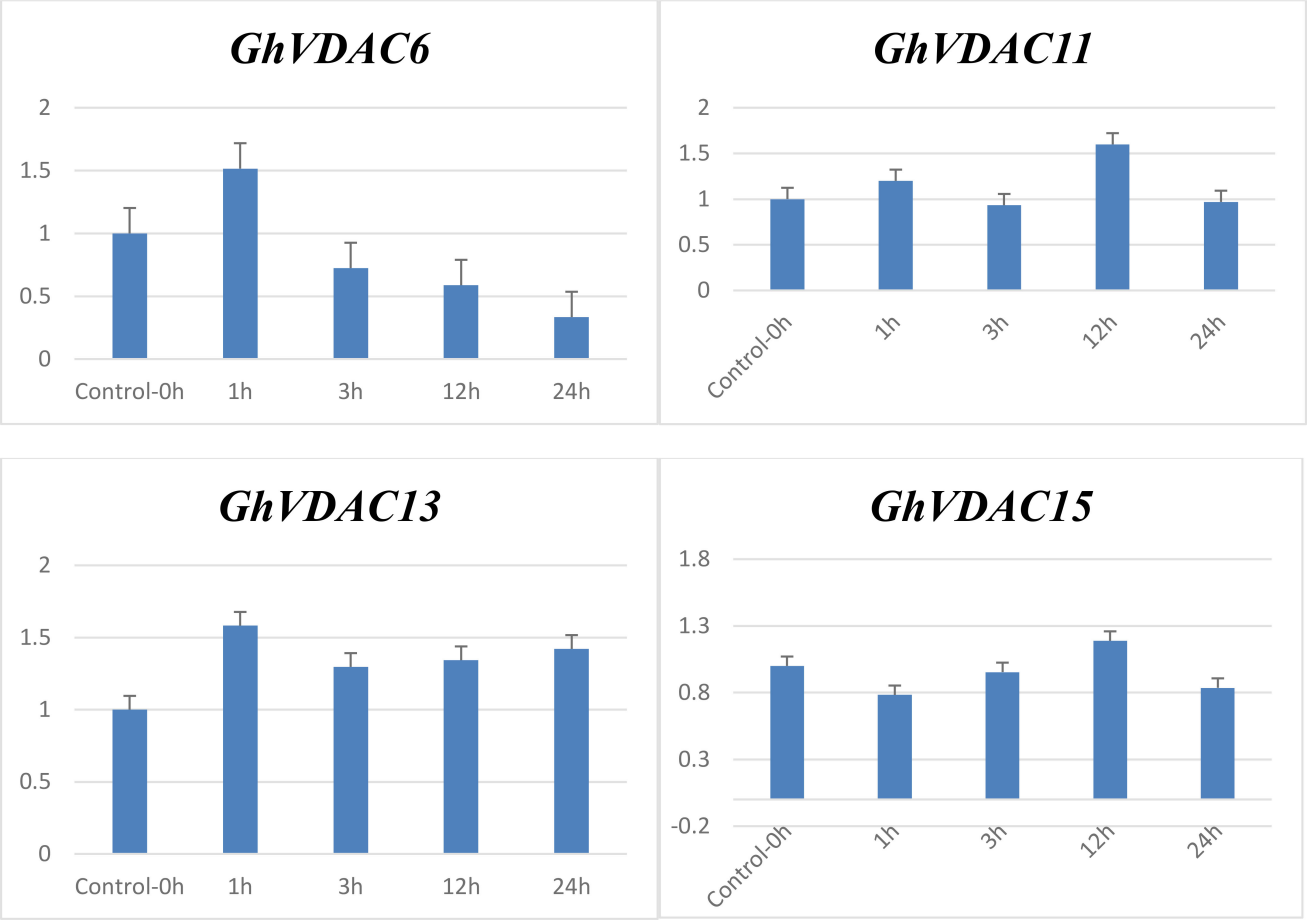
qRT-PCR expression of *GhVDAC6*, *GhVDAC11*, *GhVDAC13*, and *GhVDAC15* under drought and salt stress conditions. On X-axis are the different times intervals i.e., 0h, 1h, 3h, 12h and 24h on which the sample was taken. To check the fold expression of *GhVDAC* genes under drought and salt stresses, the control was normalized and kept constant at 1. Error bars indicate standard deviations among three independent replications. *p* < 0.05. The Y-axis represents the relative expression level of GhVDAC genes.

The qRT-PCR analysis of four candidate *GhVDAC* genes was performed, and notably, all genes were upregulated in the transcriptome data. In drought stress response, the *GhVDAC6* was minimal at 24 hours and maximum at 1 hour. Similarly, the expression level of the *GhVDAC11* gene was minimal at 3 hours and up-regulated at 12 hours. In salt stress, the *GhVDAC13* gene was minimal at 3h and up-regulated at 1 hour, whereas the *GhVDAC15* gene was minimal at 3 hours and up-regulated at 12 hours (**Figure 11**). Different primers for *GhVDAC* genes are provided in **Supplementary Table S6**.

## 4 Discussion

### 4.1 *VDAC* gene family in *G. hirsutum*

Cotton is the main source of fiber, and it is the backbone of the economy in several countries in the world (Thyavihalli Girijappa et al., 2019). But the yield of cotton around the world has been hindered due to several factors, and abiotic stresses such as heat, salt, cold, and drought are the major ones (Zhu, 2016; Zhang et al., 2022). Omics approaches have now been extensively studied and integrated to decipher the molecular mechanism leading to abiotic stress tolerance in plants (Roychowdhury et al., 2023). In plants, the resistance to these factors can only be achieved through a better understanding of the molecular mechanisms. The *VDAC* genes are found in the outer mitochondrial membrane of plants and play a role in metabolite transportation (Li et al., 2013). The study on *VDAC* genes has been done in different plants (Xu et al., 2015; Ashrafei Kolteh et al., 2022; Yu et al., 2022; Zheng et al., 2025), however, the information is scarce for the *VDAC* gene family. This study provides a better mechanism for understanding *VDAC* genes in cotton through genome-wide analysis studies.

### 4.2 *In-Sillico* Characterization of *GhVDAC*

The subcellular localization of genes is crucial for several biological processes (Ehrlich, 2002; Glory and Murphy, 2007), and WoLF PSORT analysis of the *GhVDAC* gene predicted that few genes were present in the mitochondria and cytoplasm, whereas most genes were present in the cytoplasm. *VDAC* genes are present in the cytoplasm, but they encode the proteins outside of the mitochondria. The drought resistance was affected in Arabidopsis plants due to ROS specie (Li et al., 2013). The phylogenetic tree analysis revealed that a total of 66 genes in different species were found, and these genes were divided into 4 clades. Clade I had 5 genes, Clade II had 13 genes, Clade III had 19 genes, and Clade IV had 29 genes present. The motif analysis revealed that almost all *GhVDAC* genes exhibited the same results in motif distribution except *Gh_A05G194200, Gh_A11G232500, Gh_A08G095400*, and *Gh_D07G239100.* Interestingly, *Gh_A05G194200, Gh_A11G232500*, and *Gh_D12G093700* exhibited no UTR regions.

The cis-regulatory element analysis plays a crucial role in abiotic stresses and how genes can regulate gene expression (Zhao et al., 2016). The results showed several cis-elements such as Tc-rich repeats, MBS, and LTR. Other hormone-responsive genes like P-Box, TATC-box, and ABRE (abscisic acid response) (Ezcurra et al., 2000) were also found, suggesting the involvement of the *GhVDAC* gene in response to different stresses. The CGTCA-motif and GC motif are involved in anoxic-specific inducibility (Martin-Malpartida et al., 2017).

The molecular evolutionary relationship between two different species can be determined by the synteny analysis (Cheng et al., 2012). The colinear gene pairs of *GhVDAC* genes in *G. hirsutum* L. exhibited that the gene pairs were not maintained throughout evolution in gene duplication. The specific gene association with other known proteins can be determined by PPI (Piya et al., 2014). The PPI analysis exhibited that *GhVDAC* genes had a good coefficient score and have similar functions.

### 4.3 *GhVDAC* genes under drought and salt stresses

The promoter analysis was done to determine the cis-regulatory elements of *GhVDAC* genes under abiotic stresses, and results showed that most of the cis-elements were found under the abiotic stress region. The ROS accumulation can increase under drought stress, which can lead to PCD and disrupt the plant functions (Fu and Huang, 2001; Sugimoto et al., 2014). The in-silico expression analysis of *GhVDAC* genes, the leaves RNA-seq data of control, NaCl, and drought were retrieved. The candidate genes were found via RNA-seq data, and then further qRT-PCR was done for validation of these candidate genes. The gene function can be determined by gene expression profiling (Roeber et al., 2021), and the qRT-PCR of 4 *GhVDAC* genes was done. The results showed that *GhVDAC6, GhVDAC11, GhVDAC13, and GhVDAC15* genes were up-regulated after NaCl and drought treatment. In conclusion, these genes are crucial for NaCl and drought stresses, and further research is required for the functional validation of these genes.

## 5 Conclusion

In this study of genome-wide analysis of *VDAC* genes in *G. hirsutum* L., a total of 18 genes were identified. The promoter analysis revealed that most of the genes were present in the abiotic stress region, and phylogeny analysis showed that all of the *VDAC* genes from different species were categorized in 4 clades. The candidate genes were identified through RNA-Seq analysis, and these genes were further validated via qRT-PCR. Further studies are required for the understanding deep molecular mechanisms and functional validation of these genes to develop transgenic cotton crops such as Virus-induced gene silencing (VIGS) and CRISPR-Cas technology. This study can provide the foundation for future breeding programs to develop resilient cotton crops for harsh environmental conditions.

## Data Availability

The datasets presented in this study can be found in online repositories. The names of the repository/repositories and accession number(s) can be found in the article/**Supplementary Material**.
